# Two new species of *Mediomastus* (Annelida, Capitellidae) from Tokyo Bay, Japan

**DOI:** 10.3897/zookeys.422.7501

**Published:** 2014-07-03

**Authors:** Shinri Tomioka, Eijiroh Nishi, Hiroshi Kajihara

**Affiliations:** 1 Department of Natural History Sciences, Graduate School of Science, Hokkaido University, N10 W8, Sapporo 060-0810, Japan; 2 College of Education and Human Sciences, Yokohama National University, Hodogaya, Yokohama 240-8501, Japan

**Keywords:** Taxonomy, morphology, polychaete, Pacific

## Abstract

Two undescribed species of polychaetes in *Mediomastus* (Annelida: Capitellidae) were collected from intertidal to shallow habitats in Tokyo Bay, Japan. These are *M. duobalteus* **sp. n.** and *M. hanedaensis* **sp. n.** *Mediomastus duobalteus* **sp. n.** is distinguishable from all congeners by the following characters: 1) segments 3, 4, 8–11 stainable with methyl green, 2) thoracic capillary chaetae unilimbate, 3) abdominal capillary chaetae absent, 4) paddle-like chaetae in the thorax absent, and 5) abdominal hooded hooks not flared. *Mediomastus hanedaensis* **sp. n.** is similar to *M. warrenae* Green, 2002, but differs from the latter in the shapes of the thoracic capillary chaetae and the abdominal hooded hooks, and the staining pattern with methyl green. In addition, a key to all *Mediomastus* species is provided.

## Introduction

**Figure 1.**
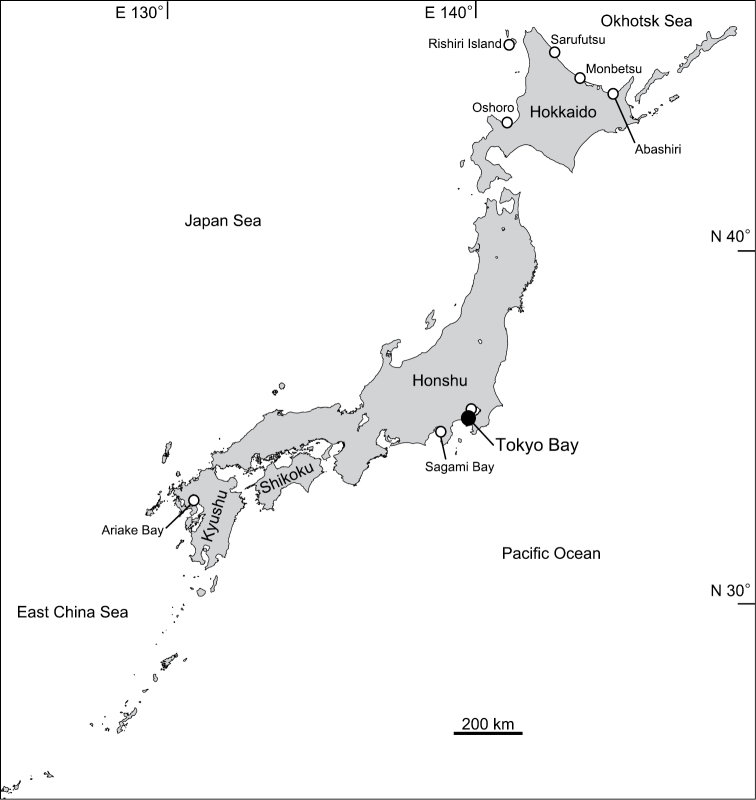
Map showing the distribution of *Mediomastus* records in Japan. Open circles, previous studies; closed circle, this study. Sources: Rishiri Island (Kato et al. 2003, Tomioka et al. 2014); Sarufutsu (Imajima 1992); Monbetsu (Tomioka et al. 2014); Abashiri (Tomioka et al. 2013); Oshoro (Tomioka et al. 2014); Tokyo Bay (Nishi and Tanaka 2007, Nishi et al. 2009); Sagami Bay (Imajima 2006); Ariake Bay (Suyama et al. 2003, Niki et al. 2006).

Capitellids in the genus *Mediomastus* Hartman, 1944 are benthic polychaete worms that occur in marine and brackish water. *Mediomastus* is distinguishable from other genera in Capitellidae by the following characters: 1) peristomium (segment 1) without chaetae, 2) thorax with 10–12 segments, 3) segments 2–5 with capillary chaetae in both rami, and 4) remaining thoracic and abdominal segments with hooded hooks (Hartman 1944, Warren et al. 1994, Green 2002). The genus was originally established for *Mediomastus californiensis* Hartman, 1944 (Hartman 1944) and now contains 14 species (Tomioka et al. 2013). In Japan, species in the genus have been reported from eight localities (Fig. 1, references therein), but only two of these records were identified to species: *Mediomastus californiensis* from Sagami Bay (Imajima 2006) and *Mediomastus opertaculeus* from Hokkaido (Abashiri, Monbetsu, Rishiri Island, and Oshoro) (Tomioka et al. 2013, 2014).

Nishi and Tanaka (2007) reported the occurrence of representatives of *Mediomastus* in Tokyo Bay but did not identify their material to species. Our capitellid specimens from Tokyo Bay were collected during an environmental assessment for the Haneda Airport re-expansion project (Nishi et al. 2009). They turned out to comprise two undescribed species, which we describe and illustrate in this paper. In addition, we provide a key to all species in *Mediomastus*.

## Materials and methods

Worms were collected from sandy mud sediment off Haneda, at the mouth of the Tamagawa River, Tokyo Bay, Japan. All specimens were fixed in 10% formalin in seawater and were later transferred to 70% ethanol after rising in deionized water. Morphological observation and methyl-green staining were performed as described by Tomioka et al. (2013). All specimens have been deposited in the Natural History Museum and Institute, Chiba, Japan. Morphological terminology follows that of Warren et al. (1994).

## Systematics

### Mediomastus duobalteus sp. n.

Taxon classificationAnimaliaScolecidaCapitellidae

http://zoobank.org/BD73215A-6470-4BE1-A439-A3F6ED585513

**Figure 2.**
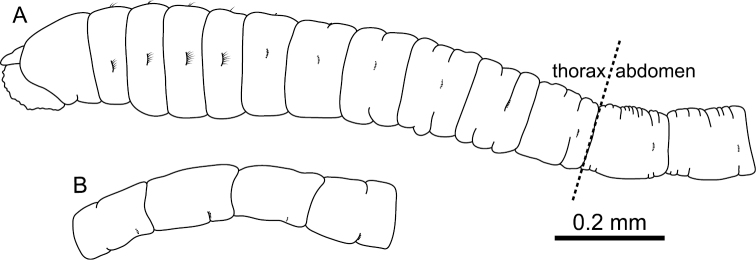
*Mediomastus duobalteus* sp. n., holotype, CBM-ZW 1088. **A** Anterior end of body, left lateral view **B** abdominal segments, left lateral view.

Figs 2 [Fig F3] [Fig F4] [Fig F5] –6

#### Material examined.

Holotype: CBM−ZW 1088, Haneda, Tokyo Bay, St. L3e-2-1 (35.52783203°N, 139.7884979°E, sandy mud bottom, incomplete, collected May 2012. Paratypes (six specimens): CBM-ZW 1089, Haneda, Tokyo Bay, St. L4e-1-1, 35.52949905°N, 139.7836609°E, incomplete; mounted on SEM stub, collected spring 2007; CBM-ZW 1090, Haneda, Tokyo Bay, St. L2b-2, 35.52531815°N, 139.7931824°E, sandy-mud bottom, 5 m depth, incomplete, some parts of body mounted on slides, remaining parts preserved in 70% ethanol, collected May 2012; CBM-ZW 1091, Haneda, Tokyo Bay, St. 07s-L4e-1-2, 35.52949905°N, 139.7836609°E, sandy mud bottom, incomplete, collected spring 2007; CBM-ZW 1092, St. 07s-L4e-1-3, 35.52949905°N, 139.7836609°E, sandy mud bottom, incomplete, collected spring 2007; CBM-ZW 1093, St. 07s-L4e-1-4, 35.52949905°N, 139.7836609°E, incomplete, collected spring 2007; CBM-ZW 1094, Haneda, Tokyo Bay, St. L4e-1-2, sandy mud bottom, incomplete, collected May 2012.

#### Description.

Holotype, anterior fragment with 73 segments; 18.0 mm in length; 0.51 mm in maximum width. Body color white in ethanol. Epithelium smooth. Nephridiopores lacking. Branchiae absent. All segments cylindrical. Sex uncertain.

Prostomium (Fig. 2A) conical, with short palpode; eversible proboscis with numerous minute papillae. Peristomium twice as long as chaetiger 1, without eyespots, achaetigerous.

**Figure 3. F3:**
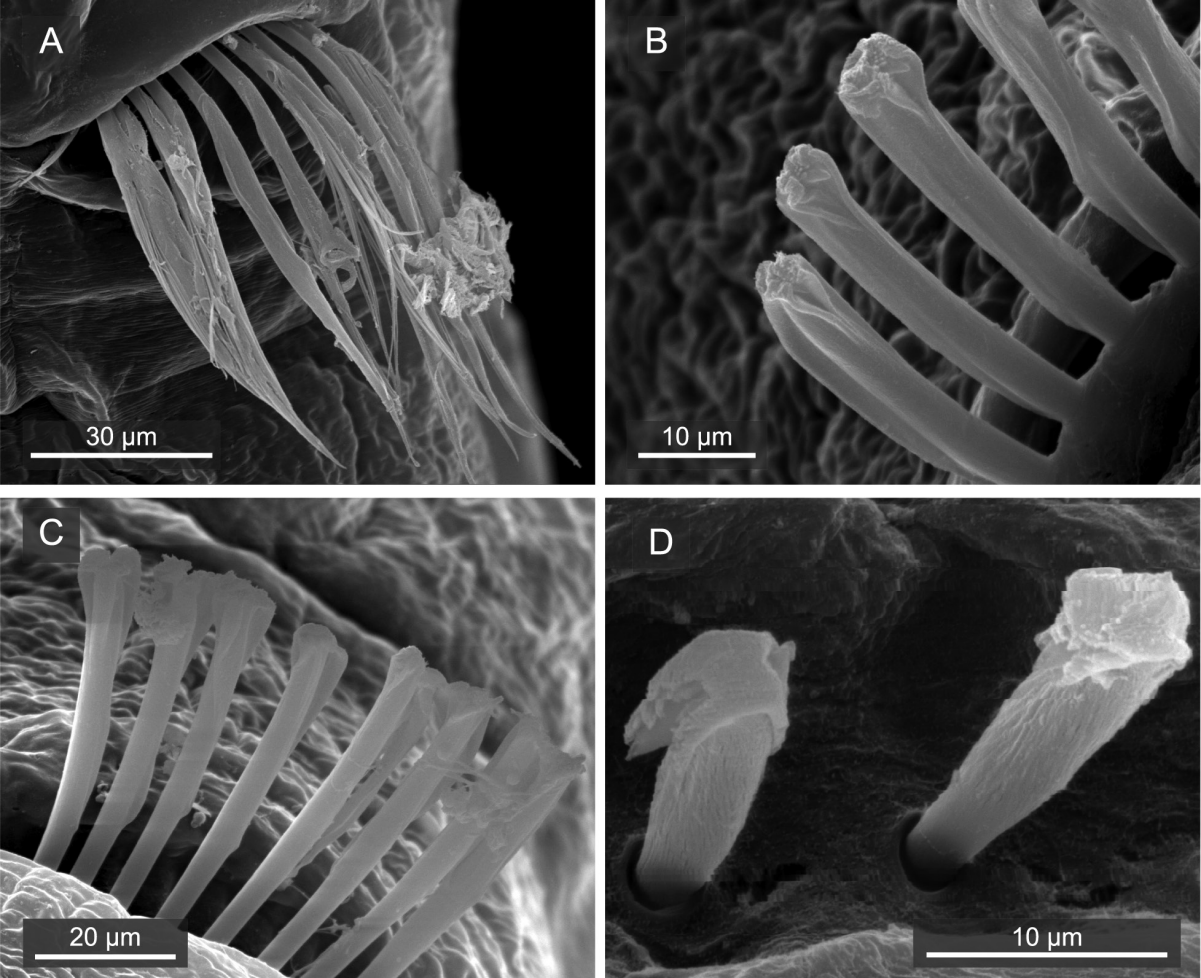
*Mediomastus duobalteus* sp. n., paratype, CBM-ZW 1089, SEM images. **A** Capillary chaetae on segment 3 **B** notopodial hooded hooks on segment 6 **C** neuropodial hooded hooks on segment 6 **D** notopodial hooded hooks on segment 13.

**Figure 4. F4:**
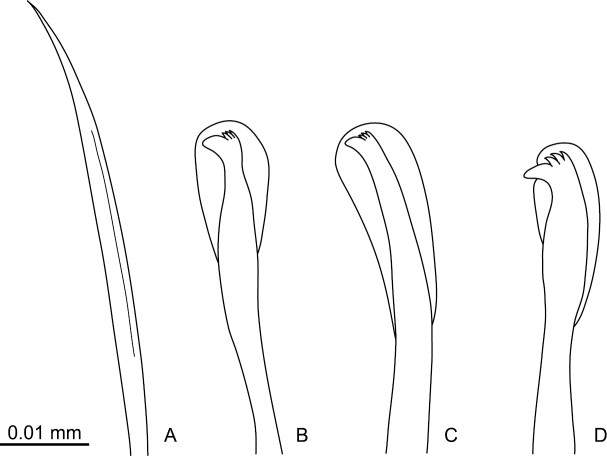
*Mediomastus duobalteus* sp. n., paratype, CBM-ZW 1090. **A** Capillary chaeta from segment 2 **B** notopodial hooded hook from segment 6 **C** neuropodial hooded hook from segment 6 **D** notopodial hooded hook from segment 20.

Capillary chaetae unilimbate, with narrow wing (Figs 3A, 4A), present on chaetigers 1–4; noto- and neurochaetae each 8–12 in number per fascicle (Fig. 3A). Chaetigers 5–10 with hooded hooks, but without paddle-like chaetae. Notopodial hooded hooks (Figs 3B, 4B) with short, stout fang and 3 fine teeth (Fig. 4B); hood with opening (Fig. 3B); shaft without constriction (Fig. 4B); shoulder indistinct (Fig. 4B); 6–10 hooks per fascicle. Neuropodial hooded hooks (Figs 3C, 4C) with short, stout fang and 3 fine teeth (Fig. 4C); hood with small opening (Fig. 3C); shaft without constriction (Fig. 4C); shoulder indistinct (Fig. 4C); 5–10 hooks per fascicle (Fig. 4C).

Abdominal segments twice as long as wide (Fig. 2B); with hooded hooks only. Hooded hooks (Figs 3D, 4D) with stout, pointed fang and 3 coarse teeth (Fig. 4D); hood with opening (Fig. 3D); shaft without constriction (Fig. 4D); shoulder indistinct; 2–6 hooks per fascicle.

Transition from thorax to abdomen marked by change in shape of hooded hooks; hooded hooks in thorax have small fang with fine, small teeth (Fig. 4B, C), while those in abdomen have stout, pointed fang; coarse and large teeth (Fig. 4D).

#### Methyl-green staining.

**Figure 5. F5:**
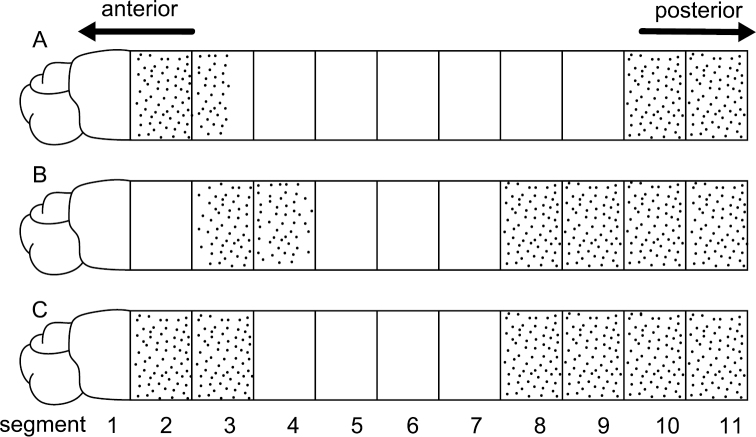
Diagram showing the three methyl-green staining patterns observed in the thorax (consisting of 11 segments) among seven specimens of *Mediomastus duobalteus* sp. n. **A** Paratype, CBM-ZW 1089 **B** holotype, CBM-ZW 1088 **C** paratype, CBM-ZW 1094.

**Figure 6.**
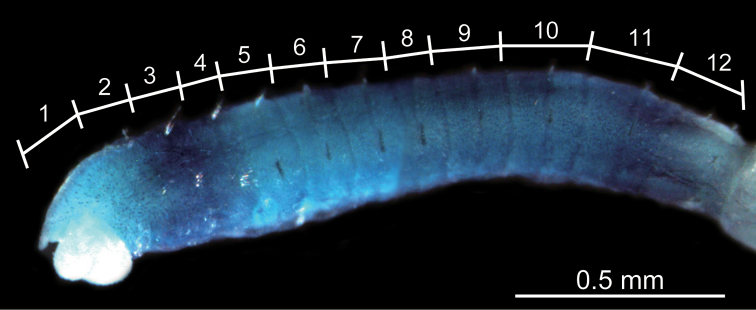
Photograph of the thorax of *Mediomastus duobalteus* sp. n., holotype, CBM-ZW 1088, showing the methyl-green staining pattern, with the segment numbers and segmental boundaries labeled.

Among seven specimens observed, methyl-green staining resulted in three patterns (Fig. 5). All patterns showed two bands of numerous, dense, minute spots: one band on segments 2 and 3, or 3 and 4; the other extending from segments 8 or 10 to segment 11. Figure 6 shows a stained worm having the pattern diagrammed in Fig. 5C.

#### Etymology.

The specific name is a noun in the nominative singular, from the Latin numeral *duo* (“two”) and the Latin noun *balteus* (“belt”), referring to the species’ diagnostic staining pattern, in which the staining pattern is two belt-like bands.

#### Remarks.

Among 14 congeners, *Mediomastus duobalteus* most closely resembles *Mediomastus warrenae*, but differs from the latter in the staining pattern with methyl green (segments 5 and 6 lack spots in *Mediomastus duobalteus* but are darkly stained post-chaetally in *Mediomastus warrenae*), the shape of the thoracic capillary chaetae (unilimbate in *Mediomastus duobalteus* vs. bilimbate in *Mediomastus warrenae*), and the shape of the abdominal hooded hooks (constriction absent in *Mediomastus duobalteus* but present in *Mediomastus warrenae*).

### Mediomastus hanedaensis sp. n.

Taxon classificationAnimaliaScolecidaCapitellidae

http://zoobank.org/CE8D8CDA-3450-4E4D-86C6-AC40DD6EA18B

**Figure 7.**
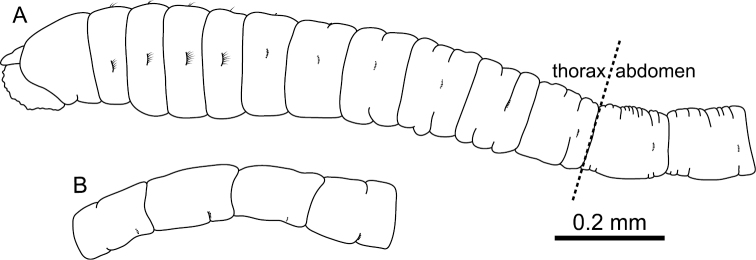
*Mediomastus hanedaensis* sp. n., holotype, CBM-ZW 1095. **A** Anterior end of body, left lateral view **B** abdominal segments, left lateral view.

Figs 7 [Fig F8] [Fig F9] [Fig F10] –11

#### Material examined.

Holotype: CBM-ZW 1095, Haneda, Tokyo Bay, St. 07s-L4e-1-c, 35.52949905°N, 139.7836609°E, incomplete. Paratypes (two specimens): CBM-ZW 1096, same collection site as holotype, incomplete, mounted on SEM stub; CBM-ZW 1097, same collection site as holotype, incomplete, cut into 5 portions, all mounted on two slides. All specimens collected spring 2007.

#### Description.

Holotype incomplete, with 27 segments, sex uncertain; 16.2 mm in length; 1.33 mm in maximum width. Body color whitish yellow in ethanol. Epithelium smooth. Nephridiopores lacking. Branchiae absent. All segments cylindrical.

Prostomium (Fig. 7A) conical, with short palpode; eversible proboscis with numerous minute papillae. Peristomium 1.2 times as wide as long, slightly longer than chaetiger 1, without eyespot, achaetigerous.

**Figure 8. F8:**
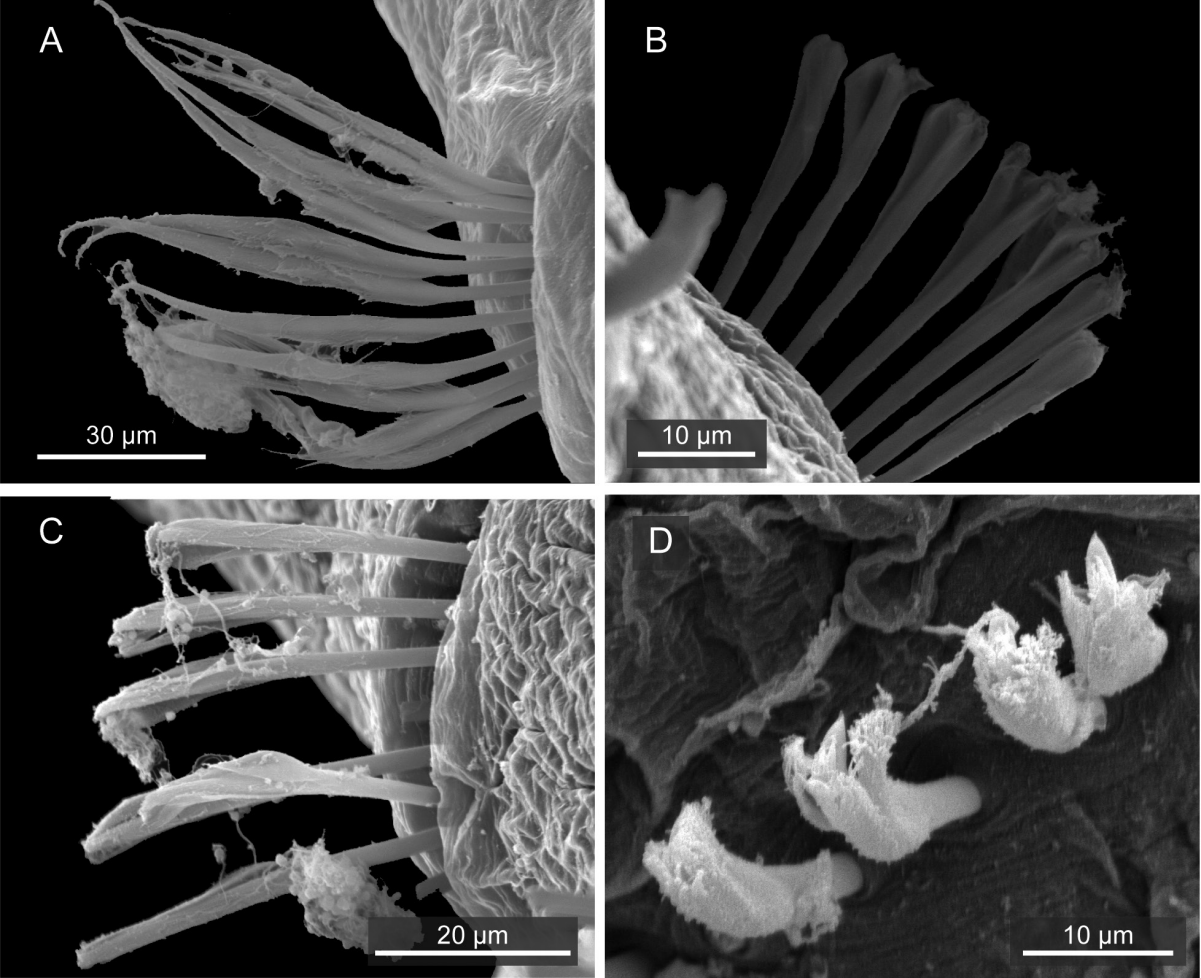
*Mediomastus hanedaensis* sp. n., paratype, CBM-ZW 1096, SEM images. **A** Capillary chaetae on segment 3 **B** notopodial hooded hooks on segment 6, with an arrowhead indicating hood opening **C** neuropodial hooded hooks on segment 6 **D** notopodial hooded hooks on segment 33.

**Figure 9. F9:**
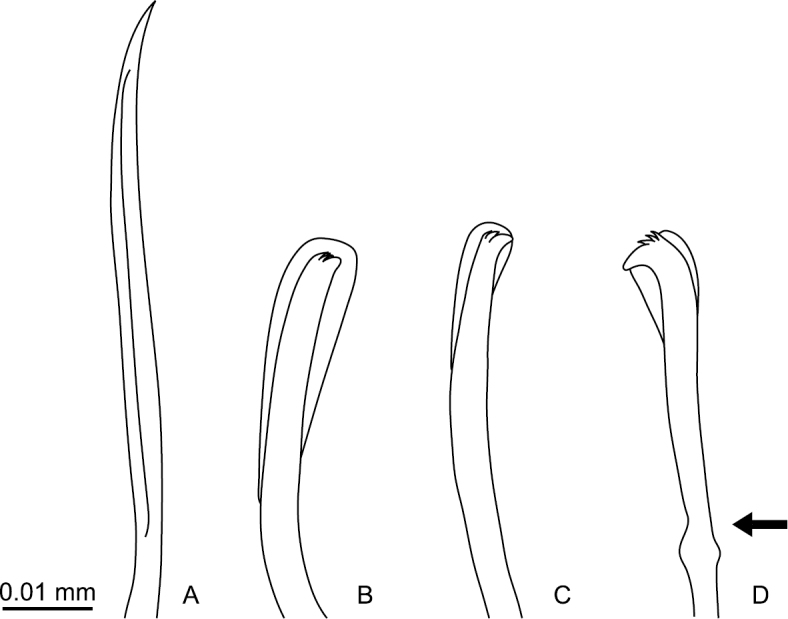
*Mediomastus hanedaensis* sp. n., paratype, CBM-ZW 1097. **A** Capillary chaeta from segment 2 **B** notopodial hooded hook from segment 9 **C** neuropodial hooded hook from segment 7 **D** notopodial hooded hook from segment 18; arrow indicates constriction.

Thoracic chaetigers biannulate. Thin, unilimbate, capillary chaetae with narrow wing (Figs 8A, 9A) present on chaetigers 1–4; noto- and neurochaetae each 8–13 in number per fascicle (Fig. 8A). Chaetigers 5–10 with hooded hooks, but without paddle-like chaetae. Notopodial hooded hooks (Figs 8B, 9B) with short, blunt fang and 6 teeth (Fig. 9B); hood with small opening (Fig. 8B); shaft not constricted (Fig. 9B); shoulder indistinct (Fig. 9B); 6–8 hooks per fascicle (Fig. 8B). Neuropodial hooded hooks (Figs 8C, 9C) with short, stout fang and 7 teeth (Fig. 9C); hood with small opening (Fig. 8C); shaft without constriction (Fig. 9C); shoulder indistinct (Fig. 9C); 4–7 hooks per fascicle (Fig. 8C).

Abdominal segments 2.5 times as wide as long (Fig. 7B), with hooded hooks only. Hooded hooks with long fang and 3 teeth (Figs 8D, 9D); fangs in abdominal hooks longer and sharper than those in thoracic hooks; opening of hood wider than that of thoracic hooks; shaft with distinct constriction (Fig. 9D); 2–5 hooks per fascicle (Fig. 8D).

Transition from thorax to abdomen marked by alteration in shape of segments (longer in abdomen), shape of shaft of hooded hooks (with constriction in abdominal hooks), and length of fang of hooded hooks (longer in abdominal hooks).

#### Methyl-green staining.

**Figure 10. F10:**
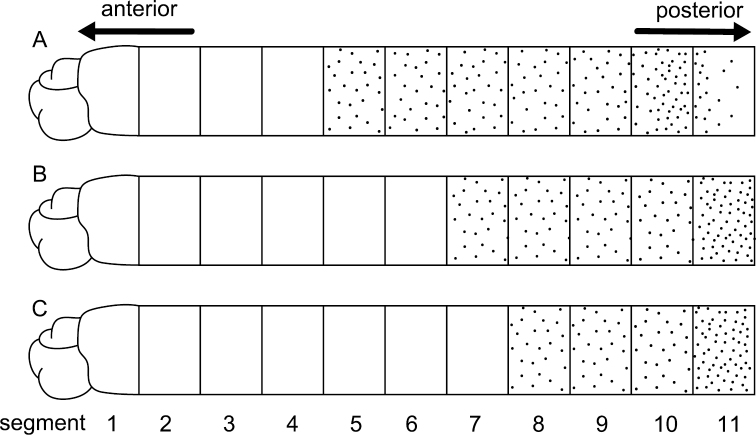
Diagram showing methyl-green staining patterns observed in the thorax (consisting of 11 segments) among three specimens of *Mediomastus hanedaensis* sp. n. **A** Paratype, CBM-ZW 1097 **B** paratype, CBM-ZW 1096 **C** holotype, CBM-ZW 1095.

**Figure 11.**
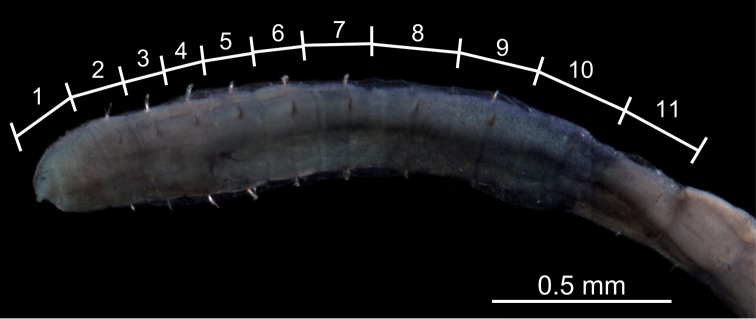
Photograph of the thorax of *Mediomastus hanedaensis* sp. n., holotype, CBM-ZW 1095, showing the methyl-green staining pattern, with the segment numbers and segmental boundaries labeled.

Among the three specimens observed, methyl-green staining resulted in three patterns (Fig. 10). Numerous minute spots sparsely and uniformly covered segments 5–9, 7–10, or 8–10; segment 10 (Fig. 10A) or 11 (Fig. 10B, C) with denser spots. Figure 11 shows the stained worm diagrammed in Fig. 10A.

#### Etymology.

The specific name is an adjective, referring to the type locality.

#### Remarks.

*Mediomastus hanedaensis* is similar to *Mediomastus warrenae* in the shape of the thoracic hooded hooks, but differs from the latter in the staining pattern with methyl green (segments 8 and 9 uniformly spotted in *Mediomastus hanedaensis* vs. post-chaetally spotted in *Mediomastus warrenae*) and in the shape of the thoracic capillary chaetae (unilimbate in *Mediomastus hanedaensis* vs. bilimbate in *Mediomastus warrenae*).

### Key to species of genus *Mediomastus*

Data compiled from Hartman (1944, 1947, 1969), Hartmann-Schröder (1959, 1962), Day (1961), Pillai (1961), Rasmussen (1973), Ben-Eliahu (1976), Warren et al. (1994), Green (2002), Tomioka et al. (2013), and this study.

**Table:** 

1	Abdominal capillary chaetae present	2
–	Abdominal capillary chaetae absent	4
2	Spine-like hooded hooks present	*Mediomastus ambiseta* (Hartman, 1947)
–	Spine-like hooded hooks absent	3
3	Thorax with unilimbate capillary chaetae	*Mediomastus setosus* Hartmann-Schröder, 1959
–	Thorax with bilimbate capillary chaetae	*Mediomastus branchiferus* Hartmann-Schröder, 1962
4	Paddle-like chaetae present	*Mediomastus acutus* Hartman, 1969
–	Paddle-like chaetae absent	5
5	Thorax does not stain with methyl green	6
–	Thoracic segments 1–4 stain with methyl green	7
–	Thoracic segments 5–11 stain with methyl green	8
6	Eye spots present	*Mediomastus fragilis* Rasmussen, 1973
–	Eye spots absent	*Mediomastus deductus* (Pillai, 1961)
7	Thorax with whip-like capillary chaetae	*Mediomastus opertaculeus* Tomioka et al., 2013
–	Thorax with unilimbate capillary chaetae	*Mediomastus duobalteus* sp. n.
–	Thorax with bilimbate capillary chaetae	*Mediomastus warrenae* Green, 2002
8	Thorax with whip-like capillary chaetae	*Mediomastus thomassini* Warren et al., 1994
–	Thorax with hook-tipped capillary chaetae	*Mediomastus capensis* Day, 1961
–	Thorax with unilimbate capillary chaetae	9
9	Nephridiopores present	10
–	Nephridiopores absent	11
10	Thoracic segments 6–10 do not stain uniformly with methyl green, resulting in striped pattern	*Mediomastus australiensis* Warren et al., 1994
–	Thoracic segments 6–10 stain uniformly with methyl green	*Mediomastus californiensis* Hartman, 1944
11	Capillary chaetae with broad wing	*Mediomastus cirripes* Ben-Eliahu, 1976
–	Capillary chaetae with narrow wing	12
12	Parapodial ridge present	*Mediomastus calliopensis* Warren et al., 1994
–	Parapodial ridge absent	*Mediomastus hanedaensis* sp. n.

## Supplementary Material

XML Treatment for Mediomastus duobalteus

XML Treatment for Mediomastus hanedaensis
