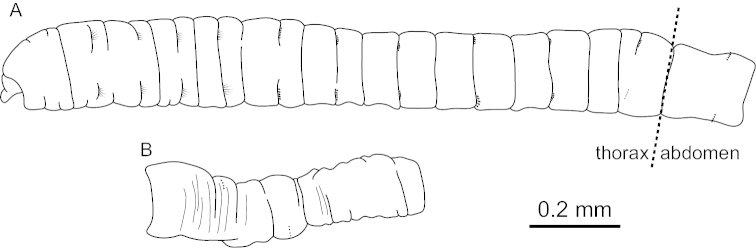# Corrigenda: Two new species of *Mediomastus* (Annelida, Capitellidae) from Tokyo Bay, Japan. ZooKeys 422: 115–126.

**DOI:** 10.3897/zookeys.463.8913

**Published:** 2014-12-12

**Authors:** Shinri Tomioka, Eijiroh Nishi, Hiroshi Kajihara

**Affiliations:** 1 Department of Natural History Sciences, Graduate School of Science, Hokkaido University, N10 W8, Sapporo 060-0810, Japan; 2 College of Education and Human Sciences, Yokohama National University, Hodogaya, Yokohama 240-8501, Japan

It has come to our attention that in the work referenced above Figure 7A, B is INCORRECT:

**Figure:**
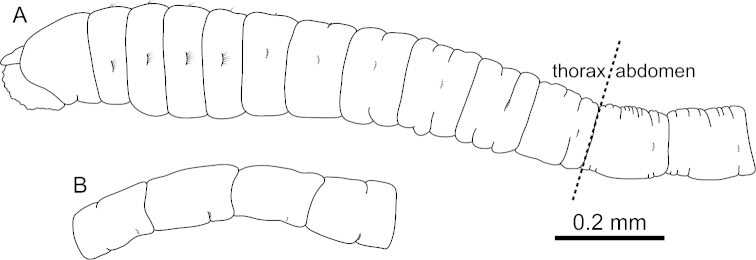


The CORRECT one is as follows:

**Figure:**